# Developing a Novel Miniature 3D-Printed TLBS with High Mechanical Efficiency and Better Controllability

**DOI:** 10.3390/mi11070662

**Published:** 2020-07-04

**Authors:** Chung-Wei Lee, Jung-Hua Chou

**Affiliations:** Department of Engineering Science, National Cheng Kung University, 1 University Road, Tainan 70101, Taiwan; jungchou@mail.ncku.edu.tw

**Keywords:** threadless ball screw (TLBS), mechanical efficiency, 3D-printed, controllability

## Abstract

This paper focuses on the development of a 3D-printed threadless ball screw (TLBS) for the applications that require miniaturization, customization, and accuracy controllability. To enhance the efficiency of the TLBS, a novel model of the TLBS for analyzing the mechanical efficiency is presented to obtain the key affecting factors. From these factors, the design parameters for fabrication are determined. For miniaturization, a novel 3D-printed one-piece preloaded structure of light weight of 0.9 g is implemented as the TLBS nut part. Experimental results show that the measured mechanical efficiency of TLBS is close to that predicted by the theoretical model with a normalized root mean square error of 3.16%. In addition, the mechanical efficiency of the present TLBS (maximum efficiency close to 90%) is better than that of the lead screw and close to the ball screw. The unique characteristic of the present TLBS is that its total torque loss is a weak function of the load, a phenomenon not observed in either the ball screw or the lead screw. This characteristic is advantageous in enhancing the controllability of accuracy at different loads.

## 1. Introduction

Rotary electric motors have been widely applied in various products and manufacturing machines. In many applications, it is essential to convert the rotary motion into linear motion. In the meantime, additive manufacturing (AM) has spread widely due to its flexibility, suitability, and being user friendly [1]. Examples include the complex geometric parts (difficult to make by traditional subtractive methods), biomedical parts requiring customization [2], micro-channels suitable for high operating pressures and micro-particle image velocimetry [3], orthoses [4], medical rapid prototyping-assisted customized surgical guides to improve the accuracy of complex surgeries [5], multi-body structures and non-assembly mechanisms [6,7,8], etc. Hence, developing a mechanism manufactured by AM technology to convert a rotary motion to a linear motion is attractive for application such as capsule robots [9] which need a customized and miniaturized actuation mechanism.

Various methods have been adopted for the conversion between rotary and linear motions, such as material deformations and lead screw mechanisms. The former can convert a translational motion into a rotational motion and can be applied in the attitude control of different devices, including those employed in space such as satellites [10]. In contrast, the latter is traditionally popular for converting the rotary motion to the linear motion. The lead screw mechanism usually consists of a nut part with inner threads and a shaft part with outer threads. While the rotary motion occurs between the nut and shaft, a relative linear motion between them will be generated. However, it has some disadvantages, such as possible vibrations produced by the sliding motion between the nut and shaft, poor mechanical efficiency due to sliding frictional losses, and backlash between the nut and shaft. For high accuracy transmission, a ball-screw mechanism is commonly adopted [11]. The ball–screw mechanism has many small balls in the gaps between the inner threads of the ball nut and the outer threads of the screw shaft. By using a ball-recycling structure to keep the balls running continuously in the gaps while the ball-screw mechanism is travelling, the conventional sliding friction is replaced by the rolling friction so that the frictional loss is greatly reduced and a high mechanical efficiency can be achieved [12,13].

In contrast, the threadless ball screw (TLBS or Rolling Ring Drive) mechanism is another alternative for overcoming the restriction of mechanical efficiency of the lead screw mechanism. TLBS differs from ball-screws by using roller bearings to replace the balls for the rolling friction effect. In 1981, Uhing [14] proposed a TLBS in which the inner edges of the roller bearings are in contact with the shaft at specific angles. While the shaft is rotating, these inner edges are driven by the shaft, tracing out an imaginary screw pattern on the shaft and causing the nut to move linearly along the shaft. That is, while both TLBS and ball screws use rolling friction to create linear motion, the former has no screw thread, no backlash, and no ball-recycling structure, whereas the latter has all three of them. Thus, the size of TLBS can be much smaller than that of ball screws.

For the development of a 3D-printed miniature mechanism, the TLBS is more suitable than the ball screw mechanism for two reasons. One is that the ball screw mechanism has screw threads in the shaft; thus, it is restricted by the manufacturing limitation of the screw thread. TLBS has no screw thread, and the lead is determined by the angle between the shaft and bearings. For the shaft with a small diameter, the lead of TLBS can be smaller than that of the ball screw mechanism due to different structures. The nut structure of the ball screw mechanism is not suitable for AM fabrication due to the complex parts of ball-recycling. However, the nut structure of TLBS is simple because no ball-recycling is used. A capsule robot for soft tissue cutting shows the success of the 3D-printed TLBS for their customized miniature actuation mechanism [9].

Modeling and analysis are essential to enhance the efficiency of mechanisms. For example, the effects of friction losses, contact angle, deformation, and preload, etc. on the performance of ball screws have been evaluated [15,16,17,18]. Similar to this effort, a novel model of the TLBS for analyzing the mechanical efficiency is presented in this paper to obtain the key affecting factors. From these factors, the design parameters for fabrication are determined. Moreover, for miniaturization, a novel one-piece preloaded structure of light weight of 0.9 g is implemented as the TLBS nut part. The validity of this approach is verified experimentally by TLBS of three different configurations of the key design factors. The experimental results showed that the measured TLBS characteristics are close to those of theoretical prediction. Furthermore, the present TLBS is more efficient than the lead screw and close to the ball screw. That is, the presently developed 3D-printed miniaturized TLBS has a high mechanical efficiency and can be applied to the applications requiring miniaturization and customization. The details of TLBS modeling, design, implementation and performance verification of the proposed novel TLBS are presented in the following sections.

## 2. TLBS Modeling

**Figure 1:**
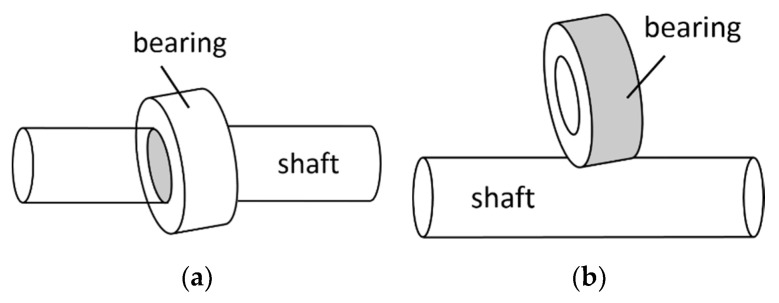
Threadless ball screw (TLBS) design types: (**a**) Bearings contact with the shaft via internal edges; (**b**) Bearings contact with the shaft via external edges.

Apart from Uhing′s design [14], with the inner edges of the roller bearings being in contact with the shaft at specific angles, the outer edges of the bearings can also contact the shaft at specific angles to create motion. Dodds and Kaminer [19] designed a TLBS with two sets of bearings. Each set has three bearings that are situated outside the nut to hold the shaft with a single-sided support. The bearings are preloaded by the nut parts with screws and springs. In contrast, the three bearings of Swanberg’s TLBS design [20] are located inside the nut and installed in the holes of the nut to hold the shaft with double-sided supports. Furthermore, Rasmussen and Hauberg [21] designed two sets of three bearings inside the nut structure so that the shaft can be held more strongly. In summary, the TLBS designs in the literature primarily have two types. One is that the bearings contact the TLBS shaft via the specially designed internal edges to ensure good contact [14], as shown in Figure 1a. The other is where the bearings contact the TLBS shaft via the external edges [19,20,21], as illustrated in Figure 1b. The latter allows designers to use common commercial bearings for the TLBS and greatly simplifies the manufacturing complexity of TLBS. Thus, the TLBS type via external edges was chosen for this study.

### 2.1. Design Parameters for Fabrication and Other Variables

**Figure 2:**
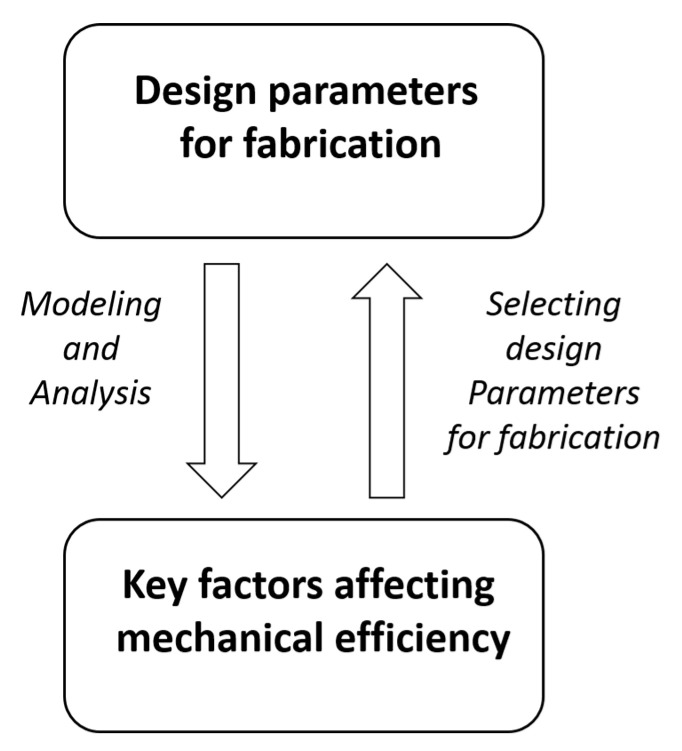
The relations between design parameters and key factors affecting mechanical efficiency.

By the procedure illustrated in Figure 2, the key factors affecting the TLBS mechanical efficiency are deduced by modeling and analysis to help select the design parameters for fabrication. The design parameters and related variables pertinent to analysis are listed in Table 1, including the lead (L), lead angle ($\theta_{l}$), and radius of the shaft (r). Two ratios (σ and α) are proposed for evaluating the TLBS characteristics, with different ranges of input torques and output forces. In this paper, the output forces of the TLBS nuts and the input torque to the shaft are all along the axial direction of the shaft of the TLBS. Hence, for simplicity, all the variables presented in the following equations are scalars. Details are described in the Section 2 and Section 3.

### 2.2. Basic Working Principle

**Figure 3:**
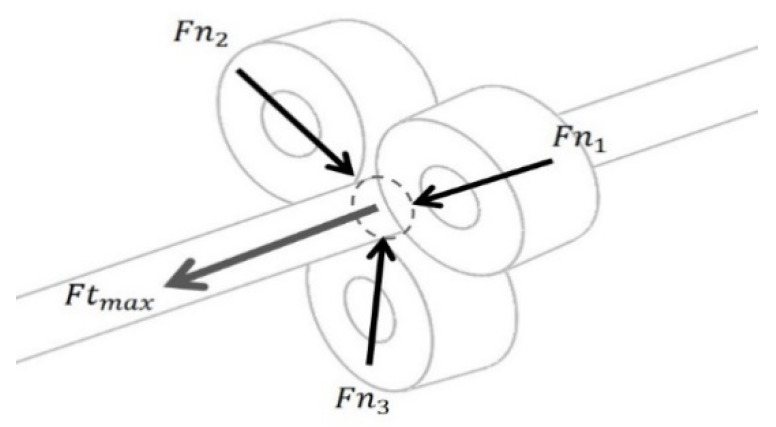
A simplified TLBS structure with normal forces and maximum output force.

**Figure 4:**
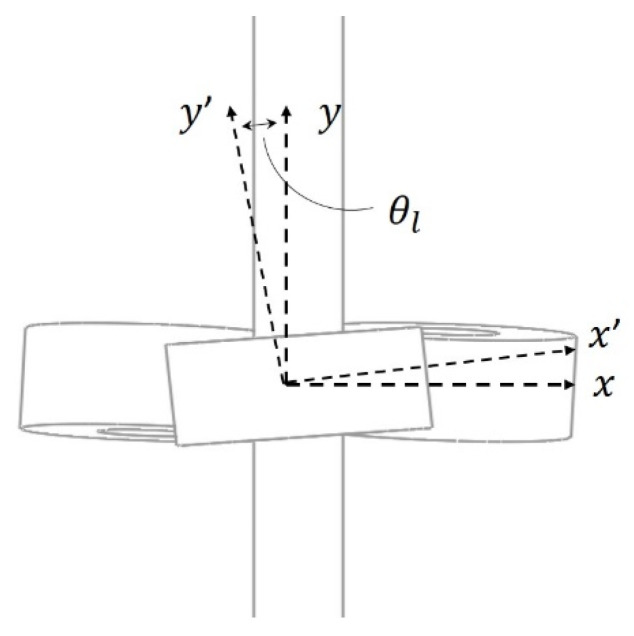
The lead angle.

The working principle of the TLBS in converting the rotary motion into linear motion is illustrated in Figure 3 using a simplified TLBS structure. The simplified structure consists of three preloaded bearings that are in contact with the shaft at specific lead angles. As the shaft rotates, the bearings trace out an imaginary screw pattern, and the nut moves the shaft due to the lead angle of the preloaded bearings and the resultant frictional forces on the shaft. The lead angle ($\theta_{l}$), as shown in Figure 4, is the angle between the shaft coordinate (x-axis and y-axis) and the preloaded bearing coordinate (x’-axis and y’-axis). The imaginary screw pattern that the bearings trace out depends on the lead angle ($\theta_{l}$), which is used for calculating the lead of TLBS by Equation (1), where r is the shaft radius.

(1) $$L=2\pi r\times \tan\theta_{l}$$

The TLBS works by utilizing frictional forces effectively. The normal forces between the shaft and the preloaded bearings, denoted by $Fn_{i}$ ($Fn_{i}=\left|{\vec{Fn}}_{i}\right|$, *i* is the *i*th bearing) provide the static friction forces as illustrated in Figure 3. The preload on TLBS is given by Equation (2) (m is the number of bearings). The maximum output force ($Ft_{max}=\left|{\vec{Ft}}_{max}\right|$) of the TLBS is equal to the net static friction forces and is obtained from Equation (3), where $\mu_{s}$ is the static friction coefficient. As the shaft rotates, the input torque to the shaft has to overcome the rolling resistance caused by $Fn_{i}$. The lost torque ($\tau_{bc}=\left|{\vec{\tau }}_{bc}\right|$)) of the bearing due to friction (referred to as friction loss torque for simplicity hereafter) is given by Equation (4), where $\mu_{r}$ is the rolling resistance coefficient.

(2) $$F_{N}≝\overset{m}{\underset{i=1}{\sum }}Fn_{i}$$

(3) $$Ft_{max}=\mu_{s}\times F_{N}$$

(4) $$\tau_{bc}=r\times \mu_{r}\times F_{N}$$

### 2.3. Analysis of Mechanical Efficiency

The friction loss torque reduces the mechanical efficiency of the screw mechanism [16,17]. With the friction loss torque of the bearings given above, Equation (5) is used to obtain the equivalent power. The left-hand side of Equation (5) is the output power of the TLBS ($Ft=\left|\vec{Ft}\right|$ denotes the output force of the TLBS); the right-hand side is the input power to the shaft ($\tau_{input}=\left|{\vec{\tau }}_{input}\right|$ is the input torque). By rewriting Equation (5) into Equation (6), the total torque loss ($\;\tau_{loss}$) includes the loss of transmission due to the lead angle and the friction loss of the bearings ($\tau_{bc}$).

(5) $$Ft\;L=\left(\tau_{input}\cos\theta_{l}-\tau_{bc}\right)2\pi \;=\left(\tau_{input}-\tau_{loss}\right)2\pi \;=\left(\tau_{input}\;\eta \right)\;2\pi$$

(6) $$\tau_{loss}=\tau_{input}\left(1-\cos\theta_{l}\right)+\tau_{bc}$$

The mechanical efficiency (η) is defined as the ratio between the input and output powers. By rewriting Equation (5) into Equation (7), the efficiency can be readily computed.

(7) $$\eta =\left[\left(\tau_{input}\;\cos\theta_{l}\right)-\tau_{bc}\right]/\tau_{input}$$

To simplify the expression, the rolling-static ratio (σ) and the output force ratio (α) defined by Equations (8) and (9) are given.

(8) $$\sigma =\;\mu_{r}/\mu_{s}$$

(9) $$\alpha \;=Ft/Ft_{max}$$

To evaluate the mechanical efficiency by the output force ratio, Equation (5) is rewritten into Equation (10). From Equations (7) and (10), the mechanical efficiency is represented as Equation (11). From Equations (1), (3), (4), (8) and (9), Equation (11) can be rewritten into Equation (12). Thus, the mechanical efficiency of the TLBS is expressed as a function of the rolling-static ratio (σ), lead angle ($\theta_{l}$), and output force ratio (α). The degrees to which these three key factors affect the mechanical efficiency will be described in Section 3.1.

(10) $$\tau_{input}=\left[\left(Ft\;L/2\pi \right)+\tau_{bc}\right]/\cos\theta_{l}\;$$

(11) $$\eta =\left(Ft\;L\cos\theta_{l}\right)/\left(Ft\;L+2\pi \tau_{bc}\right)$$

(12) $$\eta \left(\alpha \right)=\left(\alpha \;Ft{\;}_{max}L\cos\theta_{l}\right)/\left(\alpha \;Ft{\;}_{max}L+2\pi \tau_{bc}\right)\;=\frac{\left(\alpha \;\mu_{s}F_{N}2\pi r\tan\theta_{l}\cos\theta_{l}\right)}{\left(\alpha \;\mu_{s}F_{N}2\pi r\tan\theta_{l}+2\pi r\mu_{r}F_{N}\right)}\;=\left(\alpha \tan\theta_{l}\cos\theta_{l}\right)/\left(\alpha \tan\theta_{l}+\sigma \right)$$

## 3. Design and Implementation

### 3.1. Key Factors Affecting Mechanical Efficiency and Design Parameters for Fabrication

Among the three key affecting factors, the rolling-static ratio (σ) is determined by the characteristics of the selected materials of the bearing and the shaft. In this paper, the bearings and the shaft were made of steel which has a static friction coefficient ($\mu_{s}$) of 0.78 [22]. The rolling friction coefficient ($\mu_{r}$) is determined by measurements such as 0.0158 (to be described in Section 3.2). Thus, the rolling-static ratio (σ) calculated from Equation (8) is 0.0202, which is fixed in this study.

**Figure 5:**
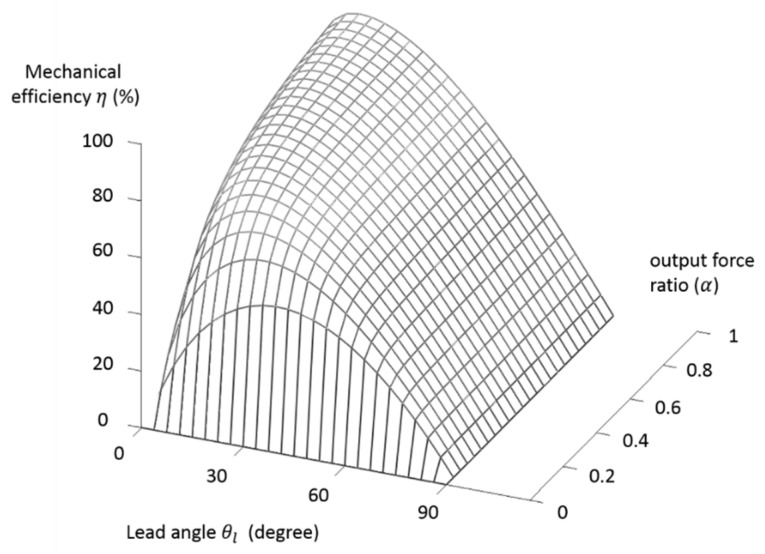
The mechanical efficiency versus different lead angles and output force ratios.

**Figure 6:**
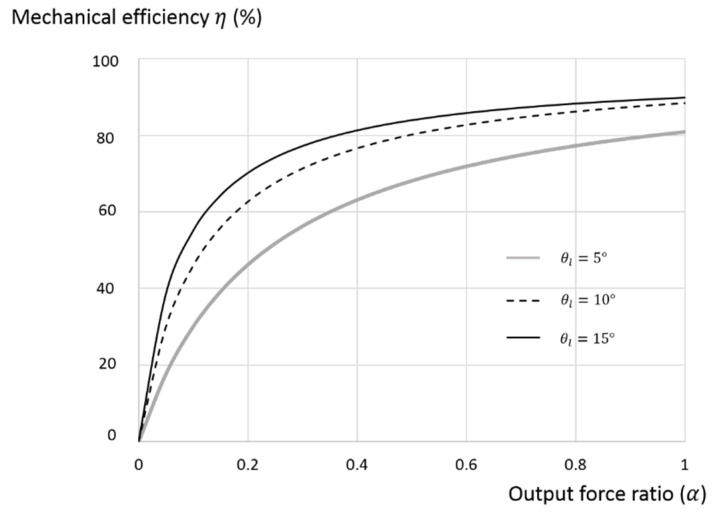
The efficiencies with lead angles of 5, 10, and 15 degrees.

The way that the lead angle and output force ratios affecting the mechanical efficiency given by Equation (12) is illustrated in Figure 5 with σ = 0.0202. The preferred range of the lead angle is from 5 to 15 degrees with the efficiency ranging from about 60 to 80% for the range of output force ratio from 0.2 to 1. To select a pertinent lead angle, the efficiencies under different output force ratios with lead angles of 5, 10, and 15 degrees are plotted in Figure 6. The lead angle of 15 degrees is selected for the present design due to its better performance compared to other lead angles for the range of output force ratios from 0.5 to 1. That is, the three key design factors that were determined are the rolling-static ratio (σ) = 0.0202, the lead angle ($\theta_{l}$) = 15°, and the output force ratio (α) = 0.5~1 with a mechanical efficiency from about 80 to 90%. After determining the lead angle, the rest of the design parameters for fabrication can be selected. The lead can also be adjusted to suit different applications by using the shaft radius (r) with the determined lead angle, as illustrated in Equation (1). For the miniaturization of TLBS, the selected shaft radius is 1 mm in this study.

### 3.2. Design and Implementation of the Novel Miniature 3D Printed TLBS

**Figure 7:**
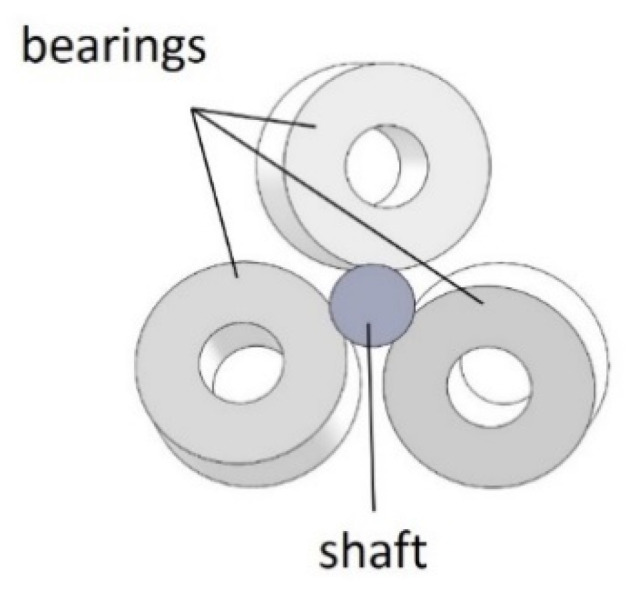
Bearing outer edges in contact with the shaft at specific angles.

The novel TLBS designed and implemented in this study includes the AM-fabricated main structures, the outer edges of the bearings in direct contact with the shaft at specific angles (shown in Figure 7), miniaturized TLBS nuts, and the utilization of commercial parts (bearings, screws and shafts) without any surface polishing. The geometry of the designed TLBS nut is very complex. As the precision of holes for bearing-mounting is critical to the transmission mechanism, the geometric error of the parts should be as small as possible. That is, the AM layer thickness should be as small as possible. Material suitability is also important, due to the constantly loaded conditions of the transmission mechanism. Many AM methods and materials can be applied to fabricate the TLBS parts [2,23,24,25]. In particular, most desktop 3D printers use the fused deposition modeling (FDM) method [2,23], which deposits the extruded molten plastic filament from a nozzle comprising a resistive heater. The extruded plastic cools down and hardens on the machine base plate. Parts are built through the successive deposition of plastic filament layers onto the base plate. Polylactic Acid (PLA) is a commonly used thermoplastic material. In comparison, Polyjet, a variant of Stereolithography (SLA), uses the UV-light-cured photopolymer (RGD525) and can produce a thin layer to achieve highly accurate, smooth and detailed models [24,25]. The features of both FDM and Polyjet methods are summarized in Table 2 [24,26,27,28,29] for comparison. In short, the materials of both FDM and Polyjet methods have similar mechanical properties. For the presently miniaturized TLBS parts, dimensional accuracy is important. Thus, the Polyjet method was selected, because it has a better resolution.

**Figure 8:**
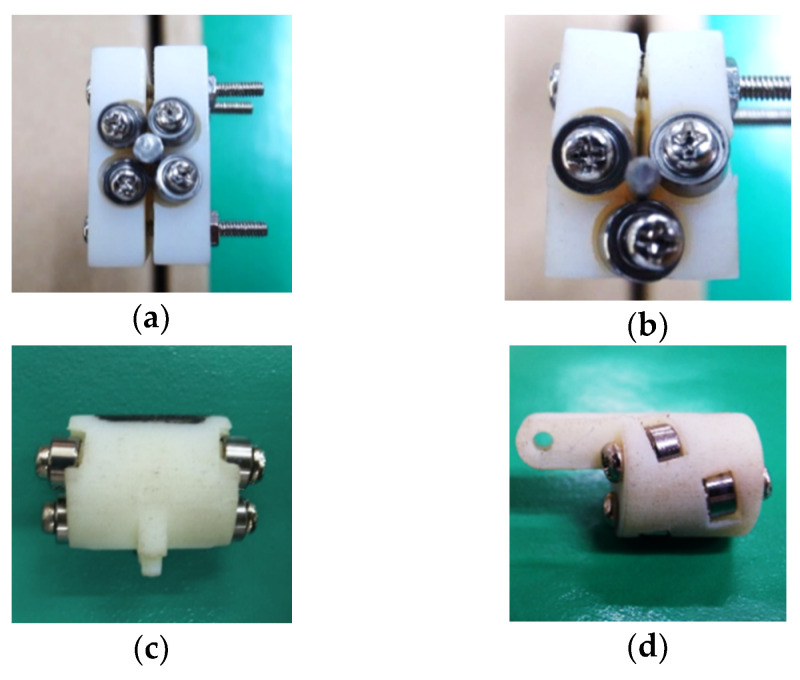
Designed and implemented prototypes of TLBS nuts: (**a**) Two-part body (weight of 5.3 g); (**b**) Single body (weight of 3.2 g); (**c**) One-piece preloaded structure with outside bearings (weight of 1.8 g); (**d**) One-piece preloaded structure with inside bearings (weight of 0.9 g).

**Figure 9:**
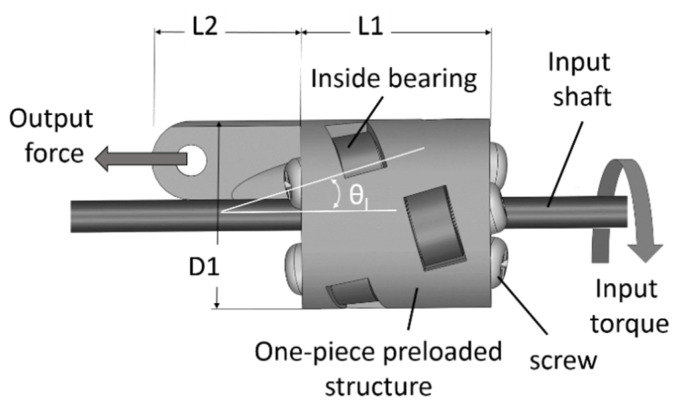
The illustration of the designed TLBS while operating.

Four designs of the TLBS nut parts were investigated. The first was a two-part body with preloaded fixtures and outside bearings. The second was a single body with preloaded fixtures and outside bearings. The third was a one-piece preloaded structure with outside bearings, similar to the design applied in the capsule robot for soft tissue cutting [9]. The last was a one-piece preloaded structure with inside bearings. Their assembled prototypes are illustrated in Figure 8a–d, respectively. They are small and light weight (0.9 g–5.3 g). In the assembly stage of prototype (a) of the two-part body design, it was difficult to assure that the stress was balanced at the preloaded fixtures, and thus broken parts were observed. For the prototype (b) of the single body design, it was easier to assemble the structure than the first design. However, stress concentration also occurred and resulted in part breaking at the bearing installation site during assembly. The one-piece preloaded structure by elastic deformation of the material, prototype (c), was designed to avoid those broken issues and performed well in the assembly stage. However, its outside bearings tended to loosen after testing runs. The prototype (d) of the one-piece preloaded structure with inside bearings performed well both in assembly and testing. This latter design has a minimum weight of 0.9 g with a small volume, and the bearings are also doubly supported to prevent loosening of the bearings. Hence, it was selected for further experiments in this study. The illustration of the designed TLBS while operating is shown in Figure 9; the output force is transmitted from the input torque of the shaft via TLBS. The associated sizes of the designed and manufactured TLBS are given in Table 3 to exhibit the accuracy of the manufactured TLBS. The geometries of nine fabricated TLBS nut parts were measured (three samples of nut parts for each lead angle). From Table 3, it can be computed that for all the quantities determined, the error of the lead angle is the largest and is close to 3.4%, whereas the errors of the others are below 1%. Thus, the fabrication method is suitable for the present application. The preloading of each fabricated TLBS is deduced by the deformation of the structure. The deformation of structure is designed by the printed parts having a smaller gap between the bearings and the shaft (the shaft radius r = 1 mm, the gap of printed part is set to 0.9 mm). The designed gap is determined by several tests, and the infilling density of the printed part is 100% for maintaining the deformation. In this study, the preload of each TLBS nut is calculated from Equation (3), with a static friction coefficient ($\mu_{s}$) of 0.78 [22] and the measured maximum output force ($Ft_{max}$). The obtained average preload (nine fabricated TLBS nut parts) is 16.06 ± 0.51 N. From the output force ratio (α), the TLBS nuts with different preloads can be compared.

To measure the rolling friction coefficient ($\mu_{r}$), the TLBS nut with the 0⁰ lead angle was also fabricated. The corresponding measured maximum output force ($Ft_{max}$) of this TLBS is 12.62 N by the force gauge. From Equation (3), the calculated preload of this TLBS ($F_{N}$) is 16.18 N, with the static friction coefficient ($\mu_{s}$) of 0.78 [22]. The measured friction loss torque ($\tau_{bc}$) of the bearings of this TLBS nut is 0.256 mNm, while its shaft can rotate at the minimum torque. Thus, the rolling friction coefficient ($\mu_{r}$) calculated by Equation (4) with r = 1 mm is 0.0158. In this study, the static friction coefficient ($\mu_{s}$) and the rolling friction coefficient ($\mu_{r}$) are assumed to be fixed because they relate only to the contact faces of commercial parts (the steel shaft and steel bearings), with a small preload of 16.06 ± 0.51 N.

## 4. Experimental Results and Discussion

### 4.1. Experiment Set-Up

**Figure 10:**
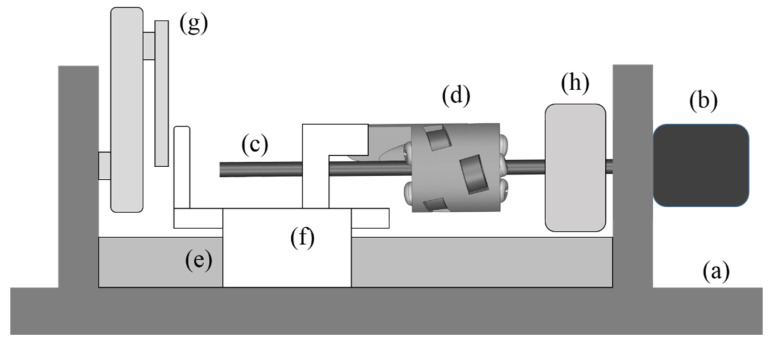
Schematic side view of the TLBS experimental setup: (**a**) Hardware base; (**b**) Motor; (**c**) TLBS shaft; (**d**) TLBS; (**e**) Linear guide; (**f**) Moving part connecting with TLBS; (**g**) Force sensor; (**h**) Torque sensor.

To confirm the presently developed TLBS, an experimental set-up was established, as shown schematically in Figure 10. The motor (b) drives the TLBS shaft (c), and the TLBS nut (d) moves linearly along the direction of the linear guide (e). The moving part (f) connecting with the TLBS nut can touch the force sensor (g) that measures the output force of the TLBS. From the torque sensor (h), the torque of the motor determines the input torque of the TLBS applied to the shaft (c).

### 4.2. Results and Discussion

**Figure 11:**
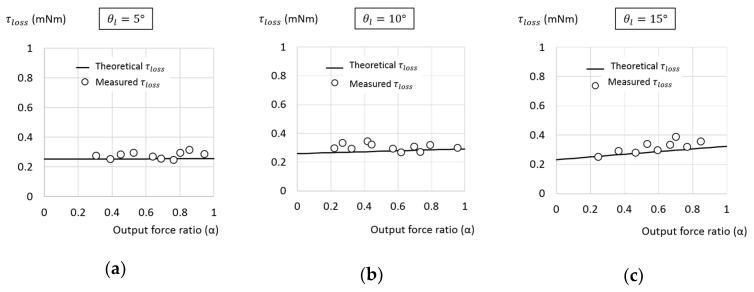
The theoretical and measured torque loss ($\tau_{loss}$) under different output force ratio for lead angle of (**a**) 5 degrees; (**b**) 10 degrees; and (**c**) 15 degrees.

To confirm the present approach, with selected σ = 0.0202 and r = 1 mm, three TLBS nuts of lead angles of $\theta_{l}$ = 5°, 1°, and 15°, were made and tested for comparison. By using the experimental setup described above, the characteristics of the TLBS were measured. The leads obtained theoretically from Equation (1) for $\theta_{l}=5{}^{\circ},\;10{}^{\circ},\;\mathrm{and}\;15{}^{\circ}$ are about 0.549, 1.107 and 1.683 mm, respectively. The corresponding measured average values of the leads are 0.54, 1.074 and 1.668 mm, close to theoretical values. The total torque loss determined from Equations (6) and (10), and measured experimentally are illustrated together in Figure 11. Both the theoretical and experimental results show that the total torque loss of the TLBS is a weak function of the output force ratio. The normalized root mean square (RMS) error of the measured total torque loss is 11.26%. The large error is caused by the small value of total torque loss (smaller than 0.4 mNm).

**Figure 12:**
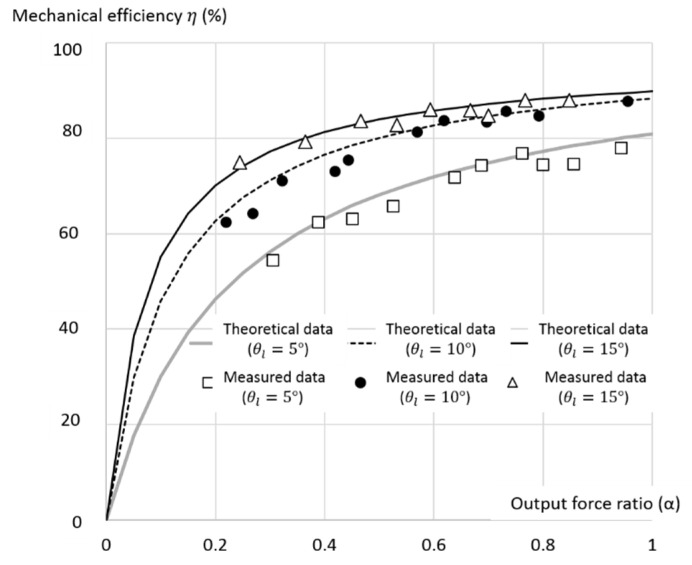
The theoretical and measured mechanical efficiency under different output force ratio with lead angle of 5 degrees, 10 degrees and 15 degrees.

The theoretical and measured mechanical efficiencies versus the output force ratio with the lead angle of 5°, 10°, and 15° are shown in Figure 12. The results indicate that a larger lead angle leads to higher mechanical efficiency, with $\theta_{l}=15{}^{\circ}$ having the highest efficiency, as expected. The experimental mechanical efficiencies are similar to those of the theoretical results, with the normalized RMS error of 3.16% demonstrating the validity of the present model, as shown by Equation (12). The result also shows that the mechanical efficiency and the output force ratio are in direct proportion, as predicted by the present model. Among the three key affecting factors of the lead angle, output force ratio, and rolling-static ratio, the lead angle is the most important because it is also the design parameter for fabricating the TLBS. Overall, the theoretical mechanical efficiencies are slightly higher than those measured due to the manufactured error of the lead angle, as described in Section 3.2. In comparison, the mechanical efficiencies of ball screws (friction coefficient of 0.01) and lead screws (friction coefficient of 0.1) with the lead angle of 15 degrees are 95% and 63%, respectively [30]. The efficiency of the present TLBS is greater than 70% for the output force ratio greater than 0.3, and from 80 to 90% for the output force ratio of 0.5 to 1. That is, the present TLBS is more efficient than the lead screw and close to the ball screw.

The commercially available minimum-sized TLBS product has the maximum length of 40 mm, and a weight of about 90 g [31]. Compared to the available commercial products, the implemented TLBS in this study is both smaller (maximum length of 21.5 mm) and lighter (weight of 0.9 g). Hence, it is suitable for applications which require miniaturization and customization. The experimental results showed that the present TLBS with the measured mechanical efficiency of 90% at larger input torques is more efficient than that of lead screws and close to that of ball screws. Furthermore, the weak function of the total torque loss to the TLBS load, as illustrated in Figure 11, is unique. This unique feature is advantageous for enhancing the controllability of accuracy due to less change in the required torque for actuation at different loads.

## 5. Conclusions

To exploit the potential of TLBS mechanisms, a novel model of the TLBS for analyzing the mechanical efficiency is presented in this paper. The analytical result indicates that the lead angle, the output force ratio, and the rolling-static ratio are the key factors affecting the mechanical efficiency of the TLBS. Among them, the lead angle is the most important factor because it is also the design parameter for the fabrication of the TLBS. The TLBS can be designed with the desired mechanical efficiency and the range of output force ratio via the present model.

For the purpose of miniaturization, a novel one-piece preloaded structure with light weight of 0.9 g was implemented as the TLBS nuts part. The corresponding performance was verified. The experimental verification results show that the measured TLBS characteristics are close to those predicted by the theoretical model. In addition, the efficiency of the present TLBS is better than that of the lead screw and is close to the ball screw. Moreover, the presently developed 3D-printed miniaturized TLBS has a high mechanical efficiency (maximum efficiency close to 90%). The unique feature of the present TLBS is that its total torque loss is a weak function of the load, a phenomenon not observed in either the ball screw or the lead screw. This characteristic is advantageous when enhancing the controllability of accuracy at different loads. Hence, the present TLBS can be applied to the applications which need miniaturization and customization, and enhance its accuracy controllability.

## Figures and Tables

**Table 1:** Descriptions of design parameters and variables.

Symbol	Description
$F_{N}$	Preload of TLBS, the total normal forces.
$Fn_{i}$	The normal forces between the shaft and the preloaded bearings, *i* is the *i*th bearing.
$F_{t}$	The output force of the TLBS nut.
$Ft_{max}$	The maximum output force of the TLBS nut.
L	Lead of TLBS; design parameter for fabrication.
r	Radius of shaft; design parameter for fabrication.
α	The output force ratio.
η	Mechanical efficiency of TLBS.
$\eta_{max}$	Maximum mechanical efficiency of TLBS.
$\theta_{l}$	Lead angle: the angle between the shaft coordinate and the preloaded bearing coordinate; design parameter for fabrication.
$\mu_{r}$	The rolling resistance coefficient.
$\mu_{s}$	The static friction coefficient.
σ	The rolling-static ratio.
$\tau_{bc}$	The friction loss torque of bearings.
$\tau_{input}$	The input torque of the shaft.
$\tau_{input\;max}$	The maximum input torque of the shaft.
$\tau_{loss}$	Total torque loss of TLBS.

**Table 2:** Features of fused deposition modeling (FDM) and Polyjet additive manufacturing (AM) methods.

**Method**	**FDM**	**Polyjet**
Machine	kingssel 3040	Objet EDEN 250
Layer thickness	50–100 μm	16 μm
Material	Polylactic Acid (PLA)	RGD525
Density	1.3 g/cm^3^	1.17–1.18 g/cm^3^
Modulus of Elasticity	3.5 GPa	3.2–3.5 GPa
Tensile Strength	50 MPa	70–80 MPa

**Table 3:** Accuracy of manufactured TLBS.

**TLBS Nut**	**Designed Dimensions**	**Measured Dimensions**
L1	12 mm	12.047 ± 0.05 mm
L2	9.5 mm	9.502 ± 0.022 mm
D1	12 mm	12.06 ± 0.051 mm
Lead angle ($\theta_{l}$)	15°	14.5 ± 0.173°
	10°	9.66 ± 0.152°
	5°	4.83 ± 0.321°
